# Epigenetic and Genetic Alterations Affect the WWOX Gene in Head and Neck Squamous Cell Carcinoma

**DOI:** 10.1371/journal.pone.0115353

**Published:** 2015-01-22

**Authors:** Seda Ekizoglu, Pelin Bulut, Emin Karaman, Erkan Kilic, Nur Buyru

**Affiliations:** 1 Istanbul University, Cerrahpasa Medical Faculty, Department of Medical Biology, Istanbul, Turkey; 2 Istanbul University, Cerrahpasa Medical Faculty, Department of Otorhinolaryngology, Istanbul, Turkey; Shanghai Jiao Tong University School of Medicine, CHINA

## Abstract

Different types of genetic and epigenetic changes are associated with HNSCC. The molecular mechanisms of HNSCC carcinogenesis are still undergoing intensive investigation. WWOX gene expression is altered in many cancers and in a recent work reduced WWOX expression has been associated with miR-134 expression in HNSCC. In this study we investigated the WWOX messenger RNA expression levels in association with the promoter methylation of the WWOX gene and miR-134 expression levels in 80 HNSCC tumor and non-cancerous tissue samples. Our results show that WWOX expression is down-regulated especially in advanced-stage tumor samples or in tumors with SCC. This down-regulation was associated with methylation of the WWOX promoter region but not with miR-134 expression. There was an inverse correlation between the expression level and promoter methylation. We also analyzed whole exons and exon/intron boundries of the WWOX gene by direct sequencing. In our study group we observed 10 different alterations in the coding sequences and 18 different alterations in the non-coding sequences of the WWOX gene in HNSCC tumor samples. These results indicate that the WWOX gene can be functionally inactivated by promoter methylation, epigenetically or by mutations affecting the sequences coding for the enzymatic domain of the gene, functionally. We conclude that inactivation of WWOX gene contributes to the progression of HNSCC.

## Introduction

Head and neck squamous cell carcinoma (HNSCC) is a heterogenous and complex disease with high incidence and mortality rate worldwide. Most of the tumors arise in the oral cavity, oropharynx, hypopharynx and larynx [1]. Despite aggressive multidiciplinary treatment approaches such as surgery, radiation and chemotherapy for patients with advanced disease the 5 year survival rate is lower than 50% and the overall 5 year survival rate is only 50–60% [2,3]. At present, the most important risk factors for the development of HNSCC remain tobacco use and alcohol consumption [4]. Relatively little information is available on the molecular mechanisms that underlie HNSCC progression. However, to improve the outcome of the disease, identification of the molecular genetic events during each step of tumor progression is crucial to understand the underlying mechanisms.

FRA16D is the second most frequent chromosomal fragile site in the whole human genome [5]. The WW-domain-containing oxidoreductase (WWOX) gene maps to a genomic region more than 1 million nucleotides long located on chromosome 16q23.1–24.1 in FRA16D [5,6]. The WWOX gene is composed of nine exons and encodes a protein of 414 aminoacids (46 kDa) that possesses two N-terminal WW-domains (responsible for protein-protein interaction) and a short dehydrogenase domain chain (SDR) exhibiting oxidoreductase activity [5].

Alterations of the WWOX gene at the genomic and expression levels have been reported in numerous neoplasias including breast [5,7,8], ovarian [9,10], esophageal [11], gastric [12,13], pancreas [14], lung [15,16] or oral cancers [17] and multiple myeloma [18,19].

Low or undetectable expression and aberrant transcription of the WWOX gene in various cancers suggest that it may act as a tumor suppressor gene [20]. Mouse knock-out experiments also support the tumor suppressor function of the WWOX gene [21,22]. Tumor suppressor genes may be inactivated by genetic and epigenetic mechanisms and their inactivation predisposes the cell to neoplastic growth.

Studies from various laboratories have revealed that the most important mechanism leading to loss of WWOX activity is genomic loss via chromosomal deletions and rearrangements [23]. Mutations in the WWOX gene are relatively uncommon and tumors with highly reduced protein levels suggest that other mechanisms in addition to deletions might affect WWOX expression. Therefore, in addition to these genetic changes, downregulation of WWOX has also been associated with epigenetic mechanisms such as promoter methylation and degradation [24,25]. More recently, it has also been shown that WWOX is silenced by miR-134 in HNSCC [25].

Despite the potential importance of the WWOX gene in carcinogenesis, genetic and epigenetic alterations of the gene have not been investigated extensively in HNSCC. Therefore, in this study we aimed to investigate the role of WWOX in HNSCC and evaluated the WWOX mutation rate and mRNA levels in HNSCC tumor tissue samples. Additionally, in order to understand the role of this gene with regard to the progression of HNSCC we assessed the association between the WWOX gene expression and mutations, promoter methylation and miR-134 levels.

## Materials and Methods

Eighty tumors and matched non-cancerous tissue samples were obtained from patients diagnosed with HNSCC and undergoing surgery in the Departments of Otorhinolaryngology, at the Cerrahpasa Medical Faculty. Cancerous and non-cancerous tissue have been identified by a pathologist. Samples with a tumor/stroma cell content greater than 70% were included in the study. Immediately after surgery, samples were divided into two portions and frozen in liquid nitrogen until nucleic acid isolation. The data on the age at diagnosis, clinical stage, histological type, and stage are shown in Table 1. The study was approved by the Cerrahpasa Medical Faculty Ethics Committee, and signed informed consent was obtained from all patients.

**Table 1:** Distributions of WWOX gene expression and methylation by clinicopathological characteristics of the patients.

**Clinicopathol. Parameters**	**Variable**	**WWOX Gene Expression**					**WWOX Promoter Methylation**				
		**Increased n (%)**	**No change n (%)**	**Decreased n (%)**	**Total n**	**p value^a^**	**Increased n(%)**	**No change n (%)**	**Decreased n (%)**	**Total n**	**p value^a^**
**Clinical stage**						**0.007**					0.397
	**Early stage (I+ II)**	9 (50)	0 (0)	9 (50)	18		7 (38.9)	5 (27.8)	6 (33.3)	18	
	**Advanced stage (III+ IV)**	9 (14.7)	1 (1.6)	51 (83.6)	61		25 (45.4)	20 (36.4)	10 (18.2)	55	
	**Unknown**	0 (0)	0 (0)	1 (100)	1	NT	1 (100)	0 (0)	0 (0)	1	NT
**Histology**						**0.005**					0.331
	**Squamous cell carcinoma (SCC)**	17 (23.6)	0 (0)	55 (76.4)	72		28 (41.8)	23 (34.3)	16 (23.9)	67	
	**Non-SCC**	1 (14.3)	1 (14.3)	5 (71.4)	7		4 (66.7)	2 (33.3)	0	6	
	**Unknown**	0 (0)	0 (0)	1 (100)	1	NT	1 (100)	0 (0)	0 (0)	1	NT
**Anatomical region**						0.583					0.606
	**Larynx**	13 (20.3)	1 (1.6)	50 (78.1)	64		25 (43.1)	19 (32.8)	14 (24.1)	58	
	**Oronasopharynx**	5 (31.3)	0 (0)	11 (68.7)	16		8 (50)	6 (37.5)	2 (12.5)	16	
**Gender**						0.070					0.741
	**Female**	4 (57.1)	0 (0)	3 (42.9)	7		2 (33.3)	2 (33.3)	2 (33.3)	6	
	**Male**	14 (19.2)	1 (1.4)	58 (79.4)	73		31 (45.6)	23 (33.8)	14 (20.6)	68	
**Age**						0.413					0.696
	**≤50**	5 (35.7)	0 (0)	9 (64.3)	14		7 (53.8)	4 (30.8)	2 (15.4)	13	
	**>50**	13 (20)	1 (1.5)	51 (78.5)	65		25 (41.7)	21 (35)	14 (23.3)	60	
	**Unknown**	0 (0)	0 (0)	1 (100)	1	NT	1 (100)	0 (0)	0 (0)	1	NT
**Histological grade**						0.698					0.866
	**Low grade (1+2)**	3 (14.3)	0 (0)	18 (85.7)	21		9 (47.3)	6 (31.6)	4 (21.1)	19	
	**High grade (3+4)**	8 (18.6)	1 (2.3)	34 (79.1)	43		16 (41.0)	15 (38.5)	8 (20.5)	39	
	**Unknown**	7 (43.7)	0 (0)	9 (56.2)	16	NT	8 (50)	4 (25)	4 (25)	16	NT

^a^ Statistical analyses were performed using the Pearson Chi-Square test (NT: Not Tested)

### RT-PCR and Real-Time Quantitative RT-PCR

Total RNA from the tissues was isolated by using the PureLink RNA Mini Kit (Ambion, USA) according to the manufacturer’s instructions. cDNAs were prepared from 350 ng of total RNA using the Ipsogen RT Kit (Qiagen, Germany). Expression levels of the WWOX gene in the tumors and non-cancerous tissue samples were analyzed by Quantitative Real Time PCR (RT-PCR) using the LightCycler 480 (Roche Diagnostics, Mannheim, Germany). PCR reactions were performed in a final volume of 20 μl containing 1×Master Mix, 300 nM gene specific primers (forward: 5’- CCCTGGAGAAGTTCACGATTC- 3’ and reverse: 5’- CAGTGAGCACACTGGTGAGATT-3’) and 200 nM hydrolysis probe (5’-6-FAM TACAAGTGTGTGCAGCCTGACTGT TAMRA-3’) which was labeled with fluorescein (FAM) at the 5′-end and with TAMRA at the 3′-end. The Glucose-6-Phosphate Dehydrogenase (G6PD) gene was used as the reference to normalize the quantification of mRNA levels. Relative mRNA levels were calculated using the 2^−ΔΔCt^ method [26].

### Sodium Bisulfite-Modification and Methylation Specific PCR (MSP)

One μg of genomic DNA was bisulfite-modified using the EZ DNA Methylation-Gold Kit (Zymo Research, Orange-USA) according to the manufacturer’s recommendations. Sodium bisulfite-treated DNA was amplified using primers specific either for the methylated or for the unmethylated DNA under the conditions as described. The sequences of the primers for the WWOX promoter were 5’-TATGGGTGTTGTTTTTTAGTT-3’ (forward) and 5’-CAATCTCCACAATATCACAACA-3’ (reverse) for the unmethylated reaction and 5’-TATGGGCGTCGTTTTTTAGTT-3’ (forward) and 5’-CAATCTCCGCAATATCGCGACA-3’ (reverse) for the methylated reaction. The PCR products were resolved by electrophoresis on a 2% agarose gel, and the ethidium bromide-stained products were analyzed using the BioID software (Vilber Lourmat, France) by calculating the volume/area ratios.

### DNA Isolation and Mutation Analysis

PCR amplifications were performed using the primers previously described [5]. The reaction conditions were: 200–300 ng of genomic DNA template, 10 pmol of each primer, 2 mM MgCl_2_, 200 μM dNTP mix, 1×PCR buffer and 1U of FIREPol DNA polymerase (Solis BioDyne, Estonia) in a 50 μl final volume. Amplifications were carried out as described previously [5]. All products were analyzed by electrophoresis on 2% agarose gels to verify the specificity of the PCR products. The reaction products were purified using the High Pure PCR Product Purification Kit (Roche, Germany) and sequencing reactions and analysis were performed on an Applied Biosystem model ABI Prism 3100-Avant Genetic Analyzer using the ABI Prism BigDye Terminator V3.1 Cycle Sequencing Kit (Applied Biosystems, CA, USA). DNA from matched non-cancerous tissues of the patients were also investigated to verify whether the detected alterations were somatic/germline mutations or polymorphisms. All of the alterations were also confirmed by a second independent PCR reaction.

### PolyPhen-2 Algorithm

To predict the possible impact of nucleotide change which resulted in an amino acid substitution the web-based PolyPhen-2 program was used. PolyPhen-2 uses eight different sequence- and three structure-based information data to predict the effect of variants using a Bayesian approach. This algorithm calculates the naive Bayes posterior probability that a given mutation will be damaging and qualitatively predicts that it will be benign, possibly damaging, or probably damaging, corresponding to posterior probability intervals, (0, 0.2), (0.2, 0.85), and (0.85, 1), respectively.

### Detection of miR-134 by Quantitative Real Time PCR

Total RNA was isolated from the tumor and non-cancerous tissue samples using the miRCURY RNA Isolation Kit-Tissue (Exiqon, MA, USA) and diluted to a final concentration of 5ng/μl for cDNA synthesis. The Universal cDNA Synthesis Kit II (Exiqon, MA, USA) was used for cDNA synthesis according to the manufacturer’s protocol. Expression levels of the miR-134 in the tumor and normal tissue samples were analyzed by Quantitative Real Time PCR (qRT-PCR) using the LightCycler 480 (Roche Diagnostics, Mannheim, Germany). The U6 small nuclear RNA was used as a reference gene for normalization. PCR reactions were performed in a final volume of 10 μl containing 1XSYBR Green Master Mix, 54 ng of each miR-134 or U6 gene-specific primers and cDNA. The reaction conditions included a polymerase activation/denaturation of 10 minutes at 95°C, 40 cycles of amplification at 95°C for 10 seconds, 60°C for 1 minute, followed by melting curve analysis, and a cooling step of 30 seconds at 40°C. Relative miRNA levels were calculated using the 2^−ΔΔCt^ method [26].

### Statistical Analysis

In the current study; WWOX and miR-134 expression levels and methylation status between tumor and normal tissue samples were compared using Chi-Square test. In addition to this, the effect of rs383362 (Ch 16: 79245820; 127 bases 3’of exon 9) alteration on the WWOX expression were compared using Kruskal-Wallis test. Clinicopathological characteristics were compared with the methylation status, expression levels and mutations of the WWOX gene using Chi-square test. Licensed version of SPSS Statistics for Windows, Version 21.0. (Armonk, NY: IBM Corp) was used for statistical analyses. p value less than 0.05 was considered to be statistically significant.

## Results

### WWOX mRNA Expression Levels

In order to determine the level of WWOX expression in HNSCC tumors, we performed quantitative analysis using real-time RT-PCR, in a cohort of 80 tumor samples and compared these with the expression levels in the adjacent non-cancerous tissue samples from the same patient. The housekeeping gene G6PD is used as an internal control for reverse transcription in all samples.

We detected the WWOX transcript in both tumor and normal tissue samples except for one tumor tissue. However, 61 of 80 tumor tissues (76.3%) showed low WWOX expression when compared to their normal counterparts (p<0.001). The mean ΔCt levels are 5.7±2.3 and 3.9±1.6 for the tumor and the normal tissue samples, respectively and the difference is statistically significant (Table 2). This indicates a 3.5-fold decrease in WWOX expression compare to the adjacent non-cancerous tissue samples.

**Table 2:** Mean expression values of the WWOX gene in tumor and normal tissues.

		**WWOX Ct (Mean±SD)**	**G6PD Ct (Mean±SD)**	**ΔCt (Mean±SD)**	**ΔΔCt**	**2^−ΔΔCt^**	**p value^a^**
	Tumor	30.9±3.5	25.1±2.5	5.7±2.3	1.81	0.29	0.001
	Normal	30.1±3.4	26.1±2.9	3.9±1.6	0	1	
**Alleles (127 bases of 3’UTR)**							
**G/G**	Tumor	33±3.3	26.8±2.8	6.2±2.4	2.37	0.19	0.299
	Normal	30.8±2.9	27±2.9	3.9±1.1	0	1	
**T/G**	Tumor	31.9±4.3	25.3±3.2	6.6±2.8	2.75	0.15	
	Normal	30.5±3.8	26.7±2.6	3.9±1.9	0	1	
**T/T**	Tumor	31.5±3.3	25.4±2.5	6.1±2.3	1.41	0.38	
	Normal	31.1±3.7	26.4±3.3	4.6±1.9	0	1	

^a^ Statistical analyses were performed using the Kruskal-Wallis test

The down-regulation in WWOX expression correlates significantly with clinical stage (p = 0.007) and histologic type (p = 0.005) but not with any other clinicopathological characteristic such as age, sex, anatomical region or histological grade (Table 1).

### Epigenetic Regulation of WWOX: WWOX Promoter Methylation and miR-134 Expression

In order to unravel whether suppression of WWOX expression in HNSCC is attributable to epigenetic mechanisms, the methylation status of the WWOX promoter was determined by MSP in the same cohort of 74 HNSCC samples. Forty (54.1%) tumor samples were partially methylated and 34 (45.9%) were unmethylated. The MSP assay shows that the WWOX gene is partially methylated in 27 (36.5%) samples but unmethylated in 47 (63.5%) of the corresponding non-cancerous tissues (S1 Fig.). Although there is no significant difference in the methylation frequency between the tumor and normal tissues, methylation of tumor tissue resultes in down-regulation of WWOX mRNA expression (Table 3).

**Table 3:** Correlation between WWOX gene expression and promoter methylation.

		**Gene Expression**		
		**Decreased n (%)**	**Increased n (%)**	**p value^a^**
**Promoter Methylation**	No change	19 (33.3)	6 (35.3)	0.006
	Decreased	8 (14)	8 (47.1)	
	Increased	30 (52.6)	3 (17.6)	
	Total	57	17	

^a^ Statistical analyses were performed using the Pearson Chi-Square test

In order to determine the level of miR-134 expression in HNSCC patients, Quantitative Real Time PCR was performed on the tumor and adjacent non-cancerous tissue samples. The U6 small nuclear RNA was used as the reference gene. miR-134 expression is detected in both tumor and normal tissue samples. We did not find any significant difference between miR-134 expression levels of the tumor and normal tissue samples. The mean ΔCt levels are 5.6±2.7 and 5.7±2.7 for the tumor and normal tissue samples, respectively (Table 4).

**Table 4:** Mean expression levels of the miR-134 gene in tumor and normal tissue.

	**miR-134 Ct (Mean±SD)**	**U6 Ct (Mean±SD)**	**ΔCt (Mean±SD)**	**ΔΔCt**	**2^−ΔΔCt^**
**Tumor**	34.1±1.7	28.5±2.3	5.6±2.7	−0.10	1.07
**Normal**	33.2±2.1	27.5±2.1	5.7±2.7	0	1

### Mutations of the WWOX Gene

**Figure 1:**
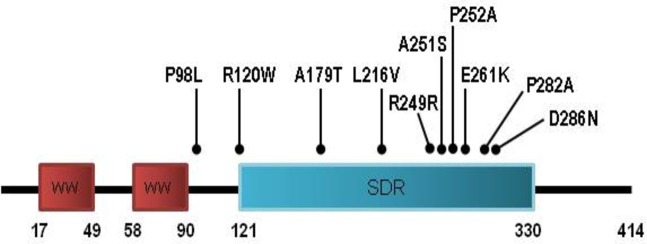
Location of coding sequence alterations of the WWOX gene.

To investigate whether the WWOX gene is mutated in HNSCC, mutation analysis was carried out in 65 of 80 tumors for which DNA for sequencing of all exons was available. Entire coding exons and the flanking intronic sequences were analyzed by PCR and direct sequencing as described previously [7]. Ten different sequence alterations in the WWOX gene were detected in primary tumors (Table 5). Two of these alterations (A179T and P282A) have been previously defined as polymorphisms. In addition to these polymorphisms we detected 7 missense mutations and one nonsense mutation (S2–S12 Figs.). The most frequent mutation is Arg120Trp with a frequency of 7.7%. On the other hand, the most frequently mutated exon is exon 7 harboring 5 different mutations. Mutations in exon 7 accounts for almost 56.3% of all mutations (Fig. 1). No mutations are present in exons 1, 2, 3, 5 and 9.

**Table 5:** Alterations in the coding sequence of the WWOX gene.

**Exon**	**Position**	**Alteration**	**n (%)**	**dbSNP ID**	**PolyPhen-2**	**% Damage vs benign**
**Exon 4**	(Arg-120→Trp) (CGG→TGG)	Heterozygous C→T transition	5 (7.7)	rs141361080	Possibly damaging	
	(Pro-98→Leu) (CCG→CTG)	Heterozygous C→T transition	1 (1.5)	rs144601717	Possibly damaging	
**Exon 6**	(Ala-179→Thr) (GCA→ACA)	Heterozygous G→A transition Homozygous G→A transition	33 (50.8) 15 (23.1)	rs12918952	Benign	
**Exon 7**	(Ala-251→Ser) (GCT→TCT)	Heterozygous G→T transversion	1 (1.5)		Benign	
	(Pro-252→Ala) (CCT→GCT)	Heterozygous C→G transversion	2 (3.1)	rs75559202	Possibly damaging	
	(Glu-261→Lys) (GAG→AAG)	Heterozygous G→A transition	3 (4.6)		Probably damaging	
	(Leu-216→Val) (CTA→GTA)	Heterozygous C→G transversion	2 (3.1)	rs7201683	Benign	
	(Arg-249→Arg) (CGC→CGA)	Heterozygous C→A transversion	1 (1.5)			
**Exon 8**	(Pro-282→Ala) (CCA→GCA)	Heterozygous C→G transversion	4 (6.1)	rs3764340	Probably damaging	
	(Asp-286→Asn) (GAC→AAC)	Heterozygous G→A transition	1 (1.5)		Benign	
**%**						23 vs 80

The possible impact of the coding sequence alterations which resulted in a different amino acid were evaluated using an algorithm utilizing sequence- and structure-based features of the protein. As a result of this analysis P98L, R120P, P252A, P282A, and E261K alterations were classified as possibly damaging while A179T, A251S, and L216V alterations were identified as benign.

In addition, 18 different sequence alterations are observed in the introns and 3′- or 5′- non-coding sequences with frequencies varying between 1.5% and 44.6% (Table 6) (S13–S14 Figs.). One of the most frequent alterations is a T to G transition at position Ch16: 79245820 (rs383362) in the non-coding, 3′-region of the gene. We observed this alteration in 38.5% and 15.4% of the tumor samples in heterozygote and homozygote form, respectively (S15, S16 Figs.). This alteration is located in a sequence which has recently been reported as a miR-134 binding sequence [24]. Therefore, in this study we also determined miR-134 expression levels in a group of the tumor tissues to investigate the role of miR-134 in WWOX expression. However, there was no statistically significant difference in the miR-134 expression levels between tumors and non-cancerous tissue samples (Table 4). We also investigated WWOX expression in the T and G allele-carrying samples to identify any possible effect of this alteration on the WWOX expression levels (Table 2). However, we did not observe a difference in the WWOX expression levels between the samples carrying the T or G alleles.

**Table 6:** Genetic alterations in the non-coding region of the WWOX gene.

**Position**	**Genetic Alteration**	**n (%)**	**Location**
5 bases 5’of exon 1 (non coding)	Heterozygous C→T transition	23 (35.4)	5’ UTR
	Homozygous C→T transition	6 (9.2)	
12 bases 5’of exon 2 (Isnp2)	Heterozygous G→T transversion	17 (26.1)	Intron 1
	Homozygous G→T transversion	1 (1.5)	
12 bases 5’ of exon 2	Heterozygous del (GA)	12 (18.5)	Intron 1
91 bases 5’of exon 3	Heterozygous G→T transversion	1 (1.5)	Intron 2
68 bases 5’of exon 3	Heterozygous C→T transition	17 (26.1)	Intron 2
	Homozygous C→T transition	20 (30.8)	
66 bases 5’of exon 3	Heterozygous T→A transversion	2 (3.1)	Intron 2
16 bases 3’of exon 4 (Isnp4)	Heterozygous A→C transversion	22 (33.8)	Intron 4
	Homozygous A→C transversion	6 (9.2)	
6 bases 3’of exon 5 (Isnp8)	Heterozygous C→T transition	29 (44.6)	Intron 5
	Homozygous C→T transition	6 (9.2)	
50 bases 3’of exon 5 (Isnp9)	Heterozygous G→A transition	10 (15.4)	Intron 5
	Homozygous G→A transition	3 (4.6)	
37 bases 3’of exon 6 (Isnp11)	Heterozygous C→T transition	12 (18.5)	Intron 6
37 bases 3’of exon 6	Heterozygous C→A transversion	1 (1.5)	Intron 6
17 bases 5’ of exon 7 (Isnp15)	Heterozygous G→A transition	2 (3.1)	Intron 6
55 bases 3’of exon 8	Heterozygous C→A transversion	4 (6.1)	Intron 8
72 bases 3’of exon 9 (non-coding)	Heterozygous C→T transition	1 (1.5)	3’ UTR
97 bases 3’of exon 9 (non-coding)	Heterozygous A→G transition	1 (1.5)	3’ UTR
	Homozygous A→G transition	1 (1.5)	
127 bases 3’of exon 9 (non-coding)	Heterozygous T→G transversion	25 (38.5)	3’ UTR
	Homozygous T→G transversion	10 (15.4)	
188 bases 3’of exon 9 (non-coding)	Heterozygous dup (AAGTA)	5 (7.7)	3’ UTR
207 bases 3’of exon 9 (non-coding)	Heterozygous del (CTAGG)	1(1.5)	3’ UTR

## Discussion

The WWOX gene is located at a common fragile region (FRA16D) and behaves as a tumor suppressor gene [5,6]. In previous studies, loss or deregulation of WWOX expression has been shown to be an important step in the development of various cancers and cancer cell lines [9,11–13,18]. Reduced expression of the WWOX gene has been reported and associated with poor prognosis and unfavorable outcome in different kinds of cancers such as breast [27], ovarian [28], non-small cell lung (NSCLC) [29], and bladder cancer [30]. In the present study, the WWOX mRNA levels were analyzed and reduced expression was observed in HNSCC. A previous study investigating WWOX expression in HNSCC has also reported reduced WWOX mRNA levels [24]. The concordance of our results with the previous study implies that inactivation of WWOX gene is a common event in HNSCC. In our study, we also assessed the correlation of WWOX expression with clinicopathological characteristics. No statistically significant association was found between WWOX expression and localization, age, gender, and histological grade except for clinical stage and histology. This indicates that the degree of WWOX inactivation may be associated with the progression of disease.

Tumor suppressor genes are silenced by genetic or epigenetic mechanisms such as mutations, deletions, hypermethylation and degradation. Hypermethylation of the promoter region of the WWOX gene has been reported as the cause of its down-regulation in various cancers [9,11,12,31]. In accordance with these reports, in our study group down-regulation of WWOX mRNA was directly correlated with promoter hypermethylation. Restoring WWOX expression by treatment of the cell lines or xenograft-bearing animals with demethylating agents has revealed that aberrant methylation of the WWOX gene is the main mechanism of its inactivation [32,33]. On the other hand, more recently Liu et al. reported that a binding sequence for miR-134 is present in the 3’-UTR region of the WWOX gene [25]. The miR-134 molecule down-regulates WWOX in HNSCC. They have shown that WWOX was down-regulated when miR-134 is up-regulated in the HNSCC tissue. WWOX expression was restored when the target sequence for miR-134 binding had been mutated. However, in contrast to these results we did not observe a difference in miR-134 levels between the down- or up-regulated tumor tissue samples. Our results indicate that there is a significant inverse correlation between WWOX expression and hypermethylation of its promoter but not with the miR-134 level. More interestingly, the miR-134 target sequence was polymorphic and in our study group 38.5% of the tissue samples were heterozygous and 15.4% were homozygous for this polymorphism. In view of this finding, we hypothesized that this alteration might affect the binding of miR-134 and WWOX expression. Therefore, we also evaluated the difference between the WWOX mRNA expression rates in the tumor samples carrying the G and T alleles but we did not observe any difference.

The frequency of mutations in the coding region of the WWOX gene has been found low in previous studies [17,34,35]. However, in this study we observed 10 different coding sequence alterations in HNSCC tumor samples. The mutation frequency was similar to the frequencies reported by our group in NSCLC [16] and breast tumors [7]. Two of these alterations (Ala179Thr and Pro282Ala) have previously been characterized as single nucleotide polymorphisms (SNPs) [5,19]. However, a recent report by Cancemi et al. [36] based on SCRATCH Protein prediction analysis suggests that the Pro-to-Ala variation causes some alterations on the secondary structure of WWOX and can affect the behavior of six different amino acids in the protein. Using the same approach they also showed that the Pro282Ala polymorphism presents a risk factor for differentiated thyroid carcinoma. In another study Gua et al. [11] have shown that the G allele elevates the risk of developing esophageal squamous cell carcinoma. Another interesting discovery was the conservation of the Proline at this position in phylogenetically distant organisms [11,36,37]. On the other hand, Pro282 is adjacent to one of the two substrate binding sites which exist on the WWOX protein [5]. These data suggest that the variant allele-282 may affect the biological function of WWOX. Therefore, this site may have functional importance altering the substrate binding capacity of the protein. We observed the Pro282Ala alteration in 6.1% of the HNSCC tumor samples. This frequency is slightly higher than those we have observed for NSCLC (4%) and lower than in breast (17.6%) tumors.

The most frequent alteration which has been observed in this study is the Arg120Trp mutation in exon 4 with a frequency of 7.69%. This alteration has been reported as a cancer-associated alteration by Paige et al. [19] and was detected in 2.4% of primary colorectal tumors and 1.8% of tumor cell lines. In our previous reports we observed this alteration in NSCLC and breast tumors with a frequency of 4 and 3.9%, respectively [7,16].

Interestingly, except one mutation (Pro98Leu) which was observed only in a single tumor tissue, all of the remaning alterations were located in exons 6–8. These exons encode the major portion of the enzymatic SDR (Short Dehydrogenase Region) domain of the protein [5]. Therefore, these alterations may affect the function of the protein and drive tumor progression.

More recently, 44 novel somatic mutations in the WWOX gene have been reported by Aldaz et al. as a result of analyzing TCGA data in various tumor types and 8 of these mutations were in HNSCC. However, we did not observe any of the alterations identified by Aldaz et al. [35] in our study group. This difference may be due to the theoretical approach and nature of the algorithm analyses. Results of database analyses need to be confirmed and validated *in vivo* using tumor fresh tissues.

In addition to coding sequence alterations we also identified 18 different alterations in the noncoding region of the WWOX gene. As discussed previously, one of the most important of these alterations is the T-G transversion in the miR-134 binding region. The other interesting alteration in the noncoding region was the −5 C to T transition in the Kozak sequence (T*C*AGCCatgG) of the gene [5]. However, we did not identify any effect of this alteration on the expression level.

We conclude that the normal function of WWOX gene may be altered by different genetic and epigenetic mechanisms. In HNSCC it is inactivated by two different hits including point mutations and methylation. Therefore, to abrogate its tumor suppressor function, its expression is reduced by promoter methylation on one side and its function is impaired by mutations on the other side. To clarify the effect of the mutations on the function of the protein further in vitro and in vivo studies need to be conducted.

## Supporting Information

S1 Fig Methylation Specific PCR analysis of the WWOX gene promoter in two cases of head and neck cancer. The presence of visible PCR products in the lanes M and U indicate the presence of methylated (347 bp) and unmethylated (347 bp) regions, respectively.
(TIF)

S2 Fig Representative chromatogram of Arg120Trp alteration (Exon 4). (TIF)

S3 Fig Representative chromatogram of Pro98Leu alteration (Exon 4). (TIF)

S4 Fig Representative chromatogram of Ala179Thr alteration (Exon 6) (Homozygous alteration). (TIF)

S5 Fig Representative chromatogram of Ala179Thr alteration (Exon 6) (Heterozygous alteration). (TIF)

S6 Fig Representative chromatogram of Ala251Ser alteration (Exon 7). (TIF)

S7 Fig Representative chromatogram of Pro252Ala alteration (Exon 7). (TIF)

S8 Fig Representative chromatogram of Glu261Lys alteration (Exon 7). (TIF)

S9 Fig Representative chromatogram of Leu216Val alteration (Exon 7). (TIF)

S10 Fig Representative chromatogram of Arg249Arg alteration (Exon 7) (Reverse strand). (TIF)

S11 Fig Representative chromatogram of Pro282Ala alteration (Exon 8). (TIF)

S12 Fig Representative chromatogram of Asp286Asn alteration (Exon 8). (TIF)

S13 Fig Representative chromatogram of a homozygous T to G transition observed in 3’-UTR of the WWOX gene (Reverse strand). (TIF)

S14 Fig Representative chromatogram of a heterozygous T to G transition observed in 3’-UTR of the WWOX gene (Reverse strand). (TIF)

S15 Fig Representative chromatogram of a homozygous C to T transition observed in 5’-UTR of the WWOX gene. (TIF)

S16 Fig Representative chromatogram of a heterozygous C to T transition observed in 5’-UTR of the WWOX gene. (TIF)
