# Supplementary figures and images for: Epigenetic and Genetic Alterations Affect the WWOX Gene in Head and Neck Squamous Cell Carcinoma
Source: PLoS One. 2015 Jan 22;10(1):e0115353. doi: 10.1371/journal.pone.0115353 (PMC4303423; doi:10.1371/journal.pone.0115353)

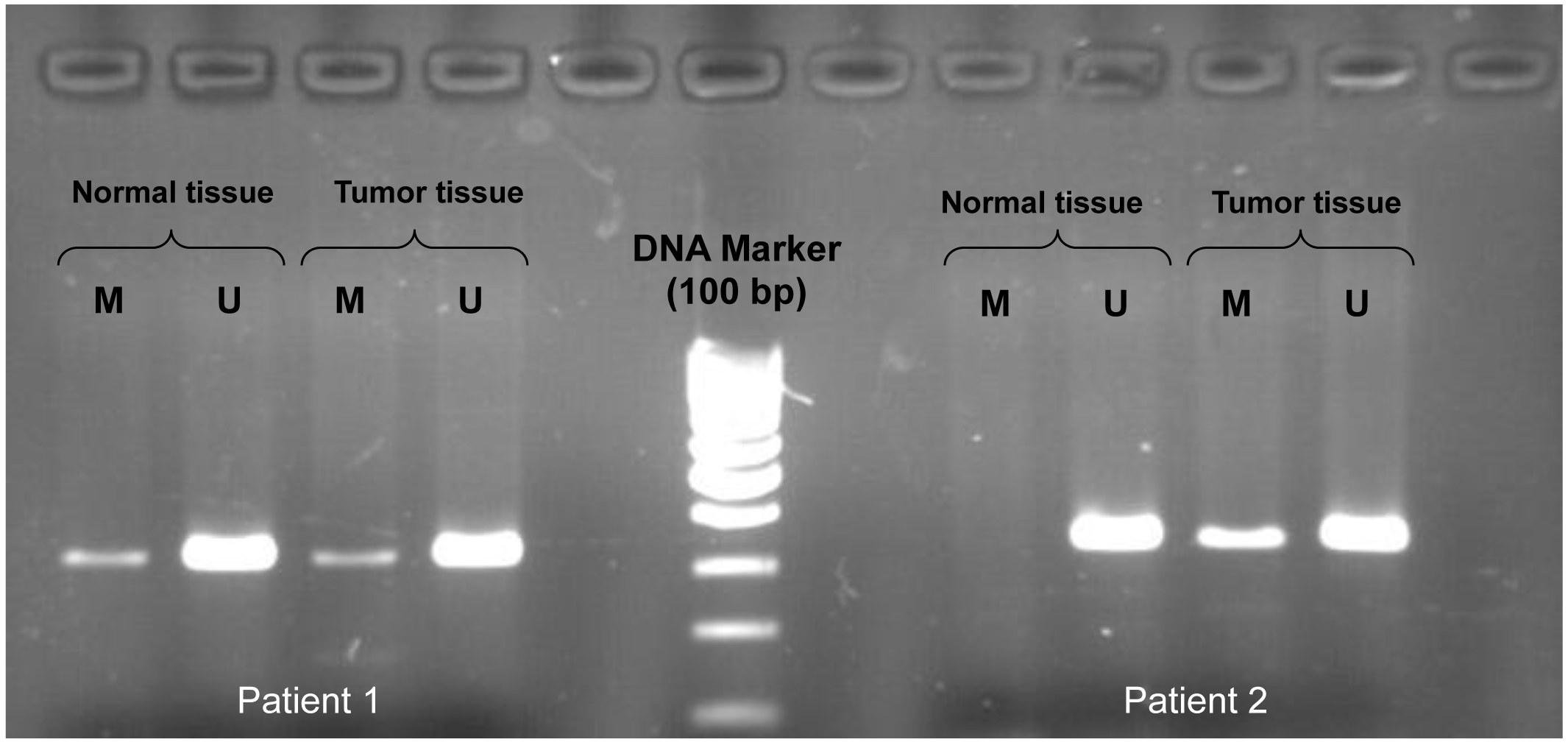

Supplement: S1 Fig — The presence of visible PCR products in the lanes M and U indicate the presence of methylated (347 bp) and unmethylated (347 bp) regions, respectively. (TIF) [file pone.0115353.s001.tif]

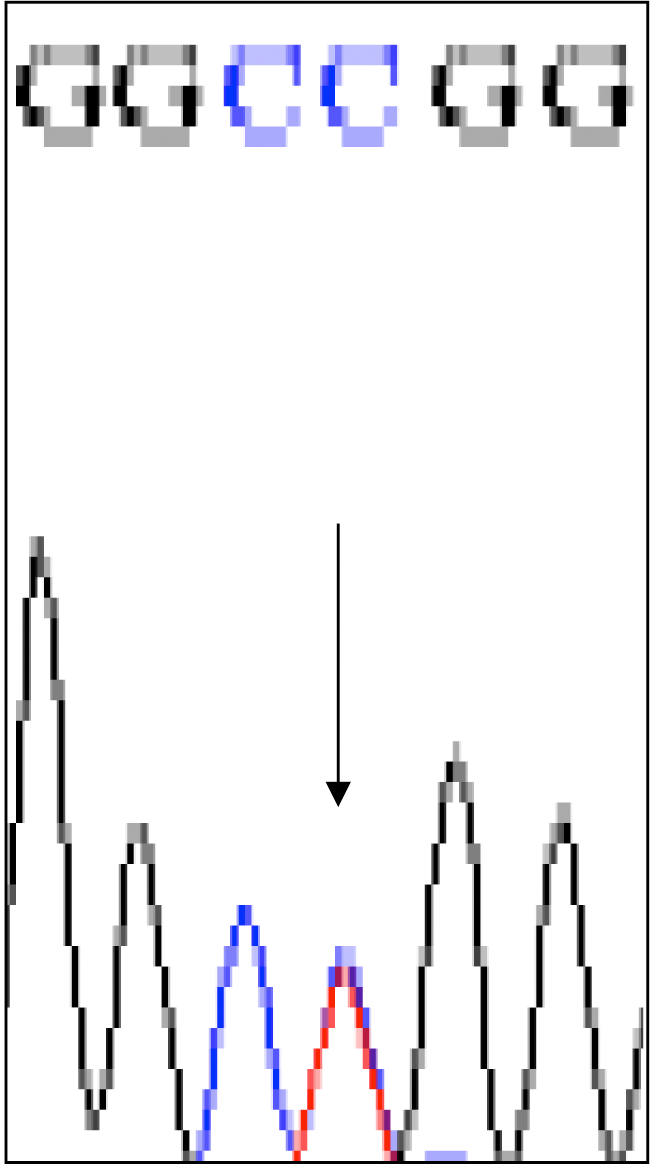

Supplement: S2 Fig — (TIF) [file pone.0115353.s002.tif]

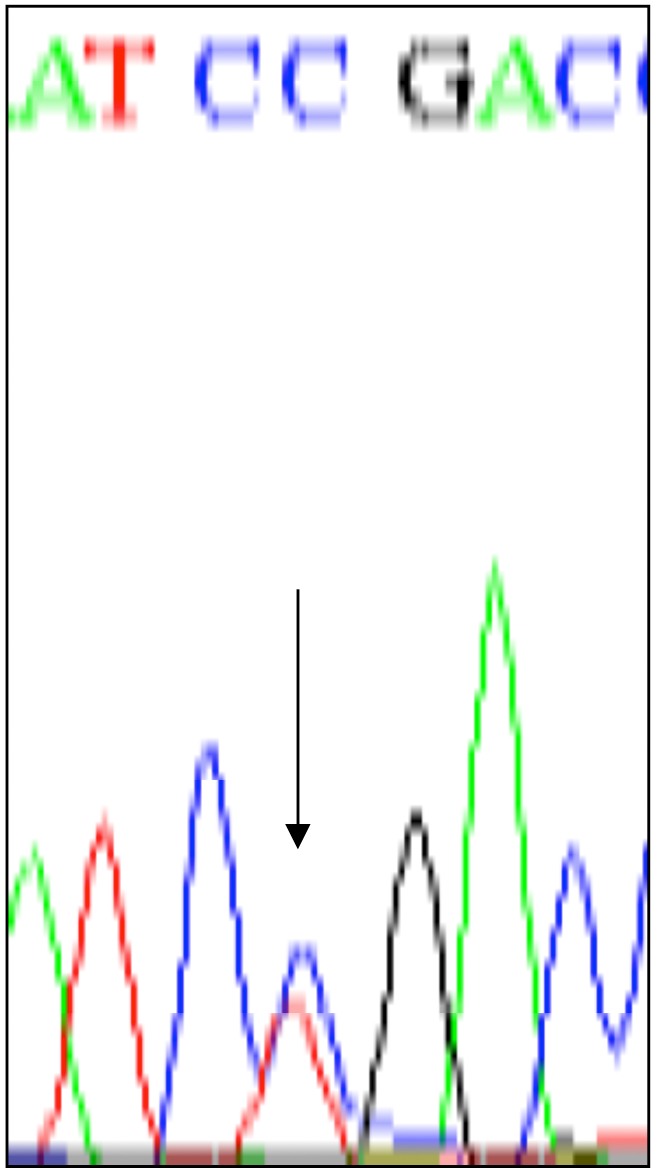

Supplement: S3 Fig — (TIF) [file pone.0115353.s003.tif]

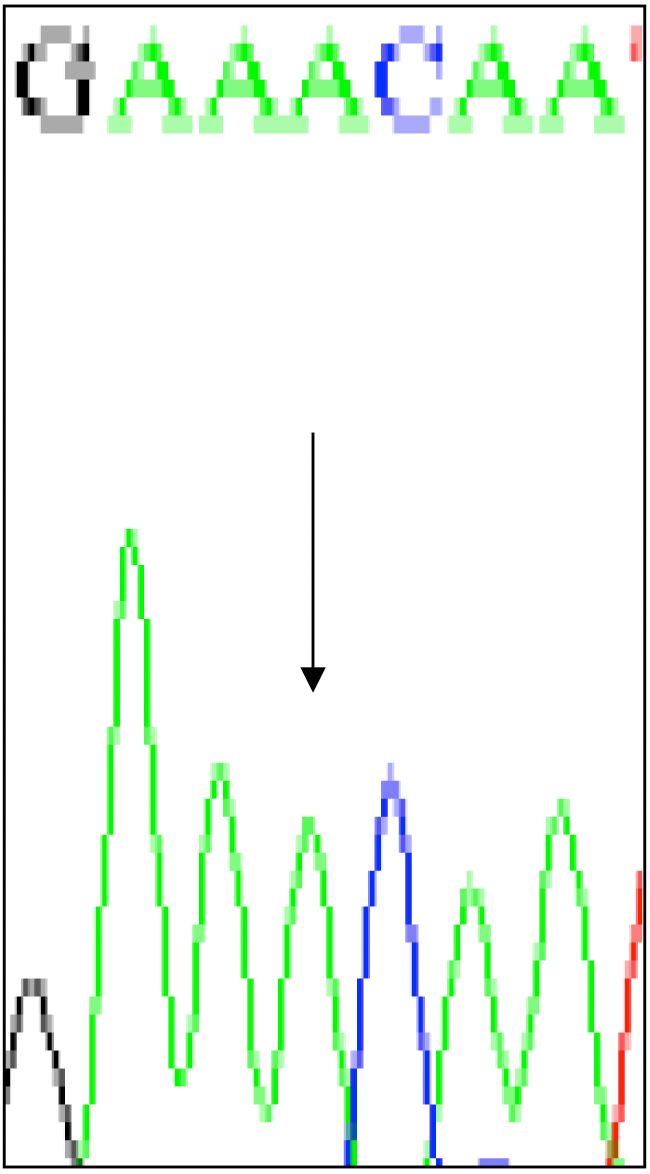

Supplement: S4 Fig — (TIF) [file pone.0115353.s004.tif]

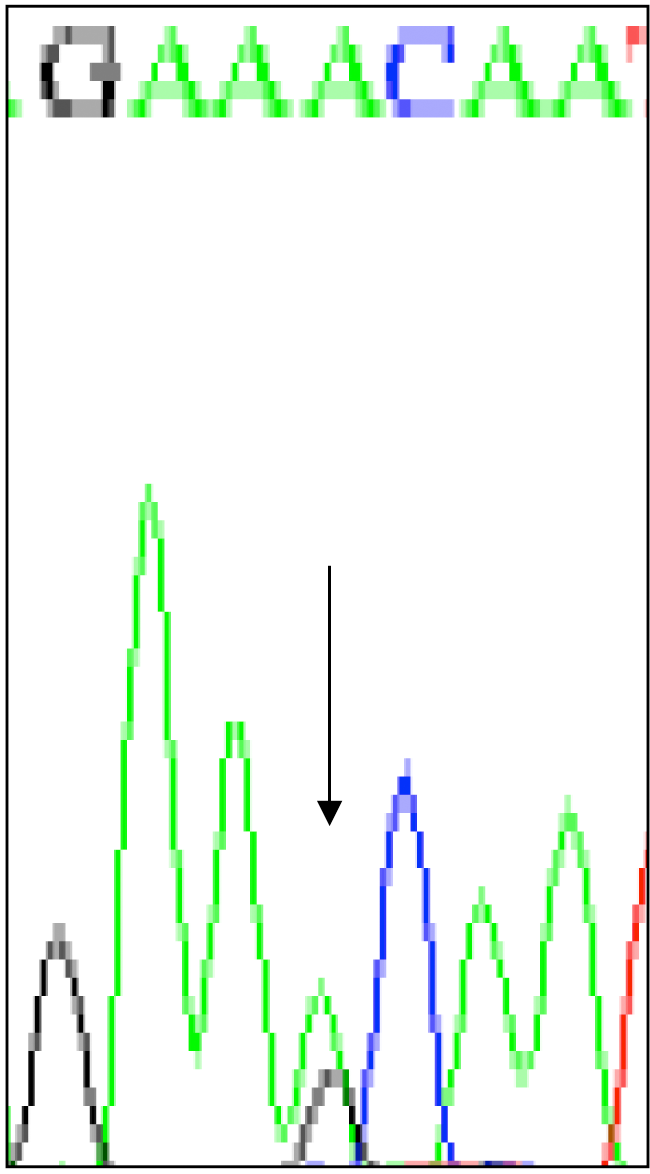

Supplement: S5 Fig — (TIF) [file pone.0115353.s005.tif]

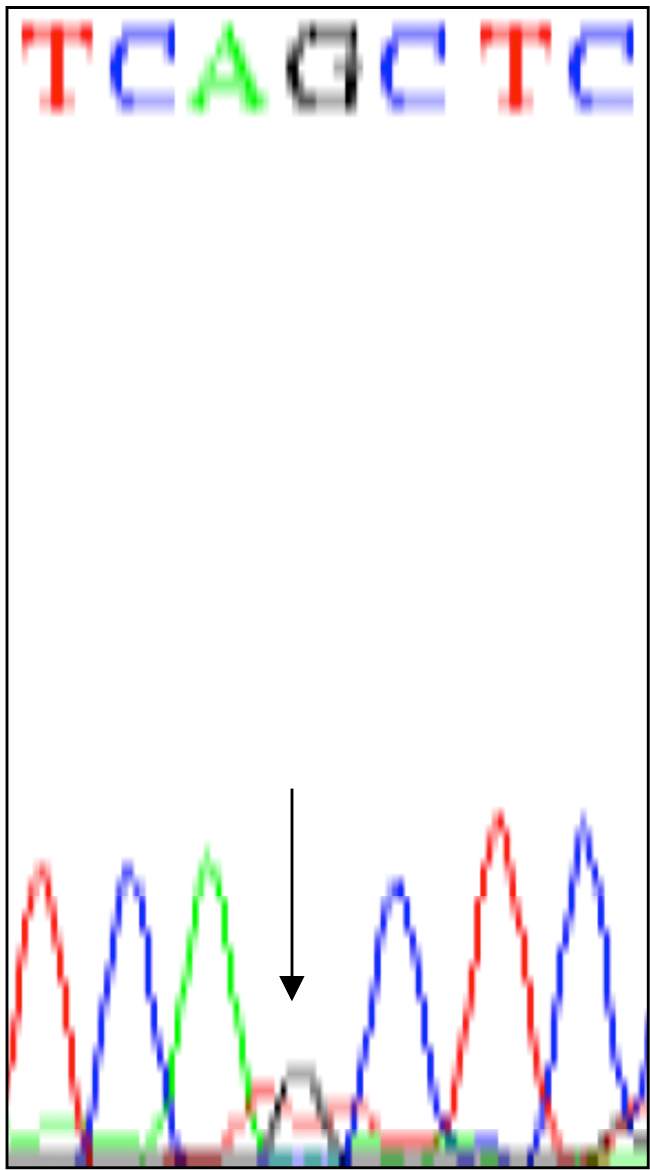

Supplement: S6 Fig — (TIF) [file pone.0115353.s006.tif]

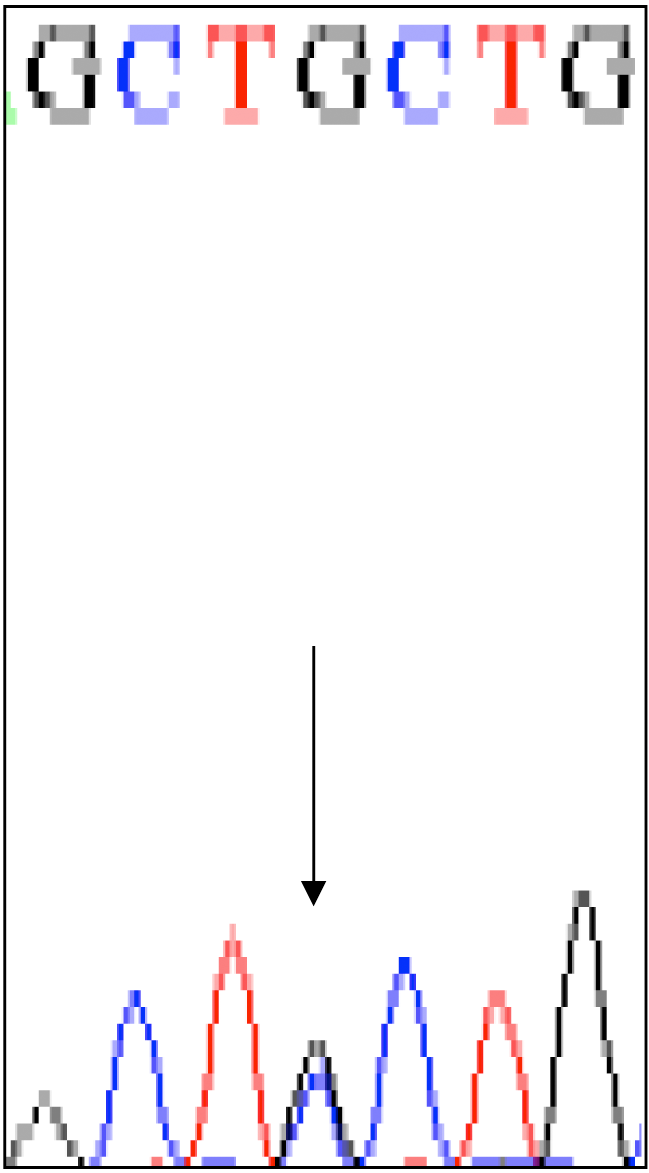

Supplement: S7 Fig — (TIF) [file pone.0115353.s007.tif]

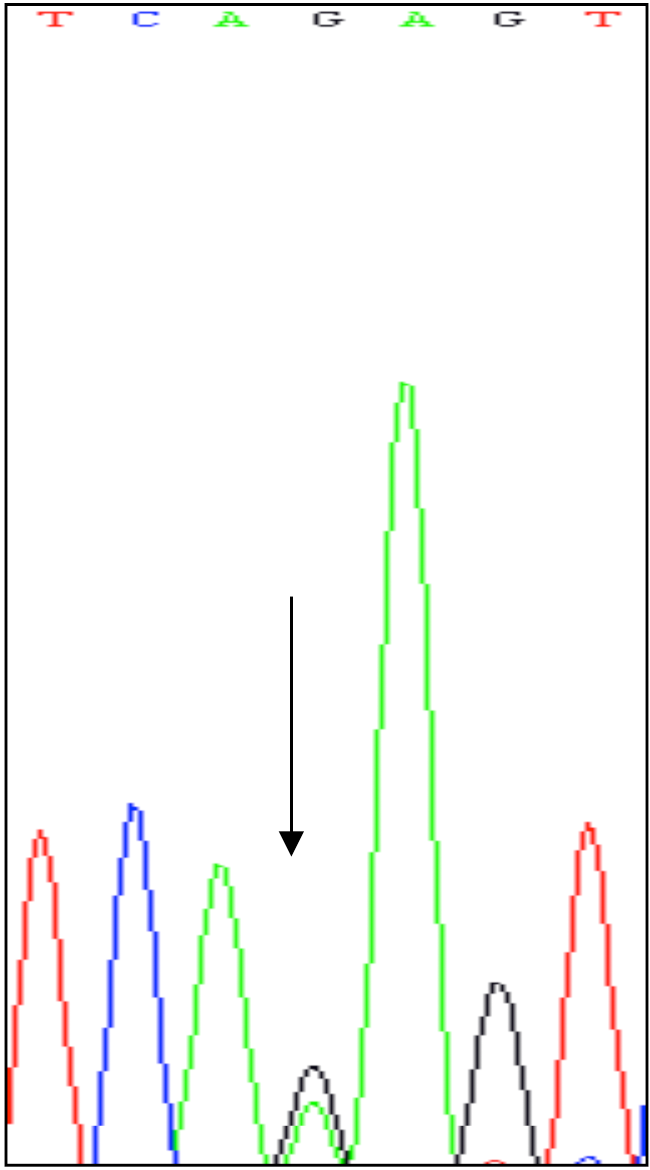

Supplement: S8 Fig — (TIF) [file pone.0115353.s008.tif]

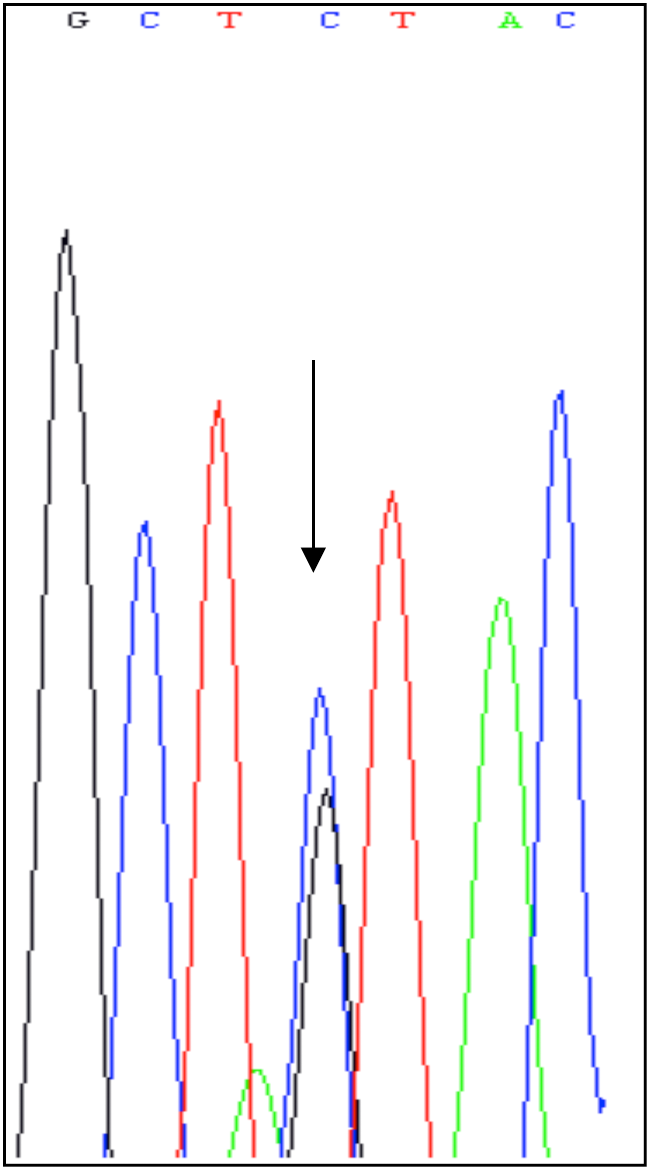

Supplement: S9 Fig — (TIF) [file pone.0115353.s009.tif]

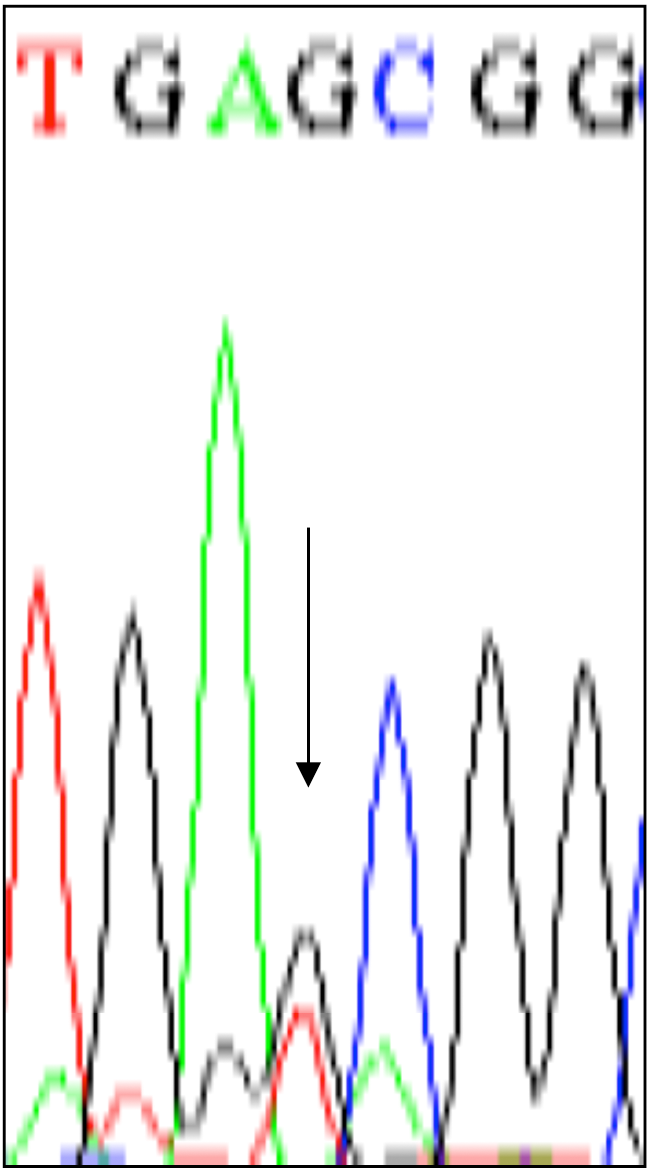

Supplement: S10 Fig — (TIF) [file pone.0115353.s010.tif]

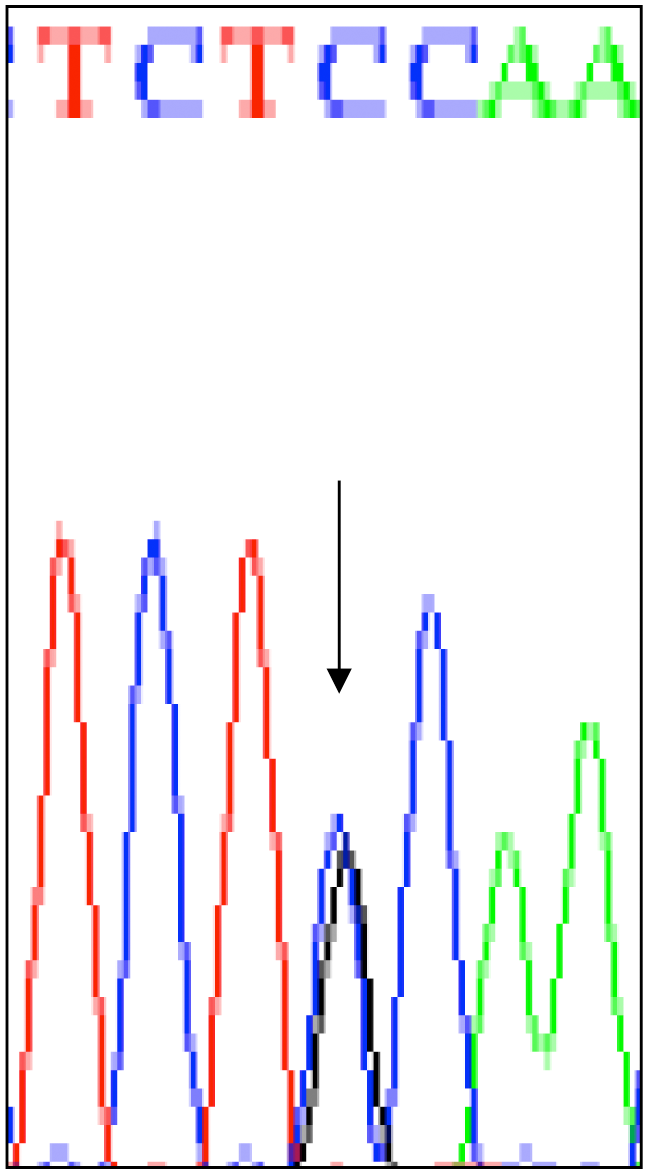

Supplement: S11 Fig — (TIF) [file pone.0115353.s011.tif]

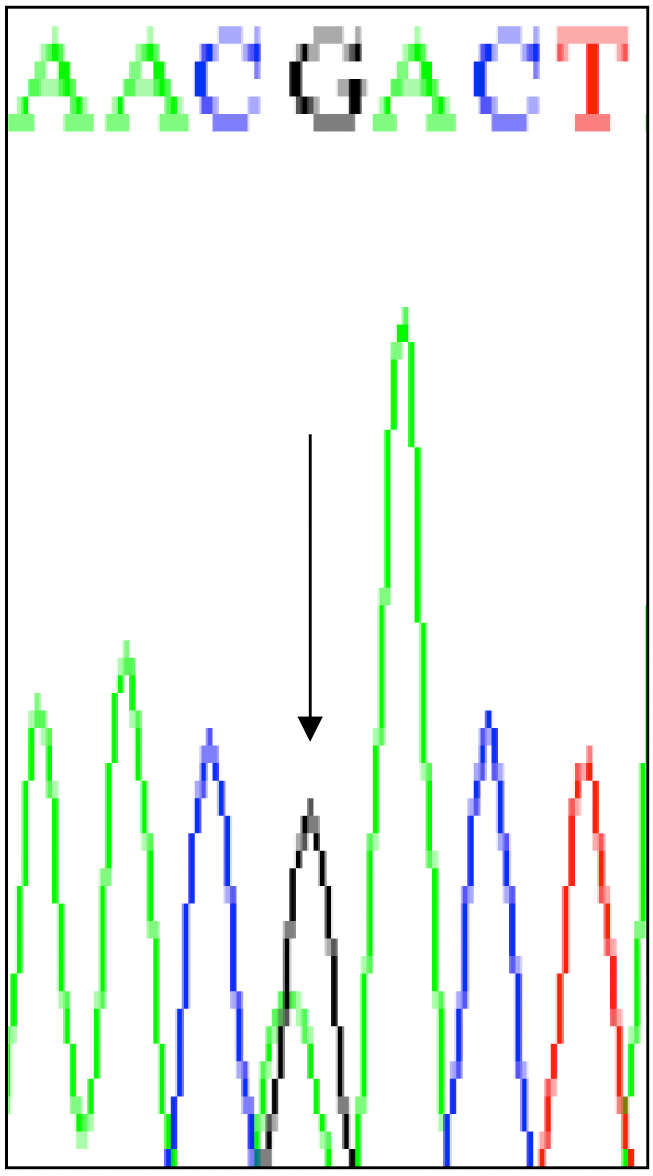

Supplement: S12 Fig — (TIF) [file pone.0115353.s012.tif]

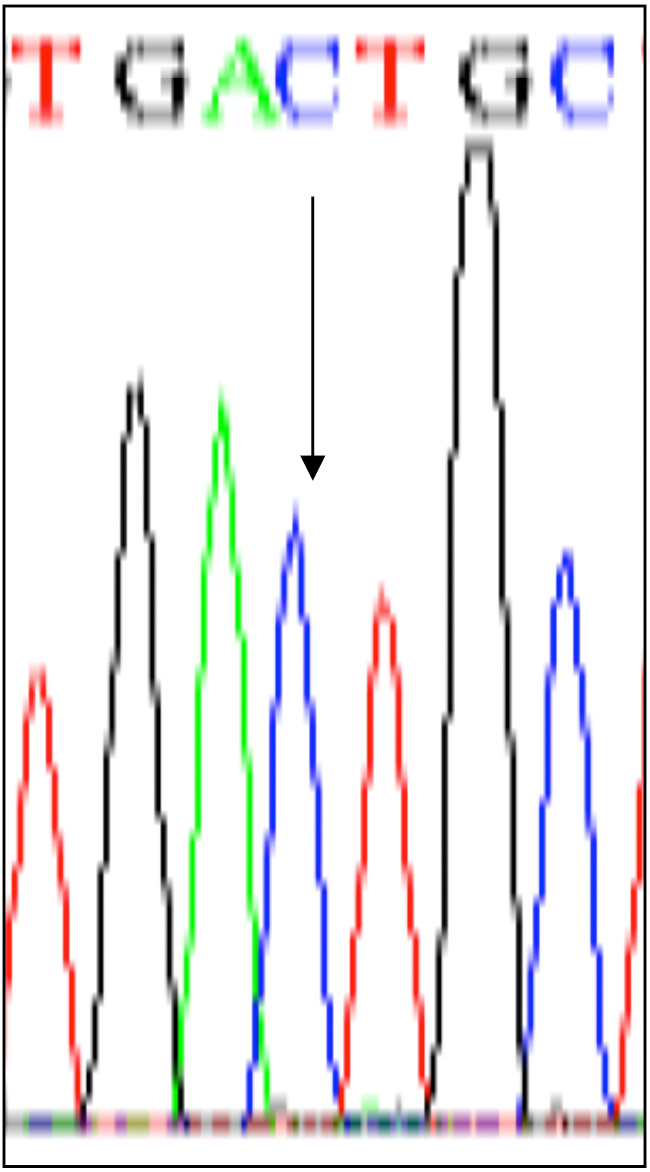

Supplement: S13 Fig — (TIF) [file pone.0115353.s013.tif]

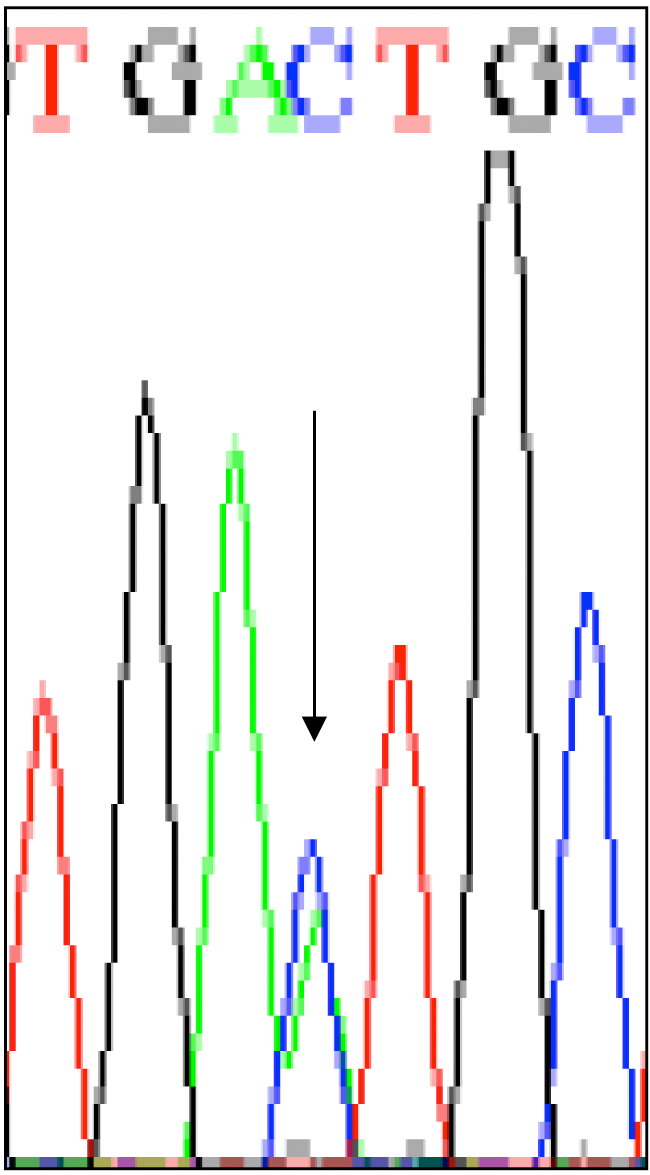

Supplement: S14 Fig — (TIF) [file pone.0115353.s014.tif]

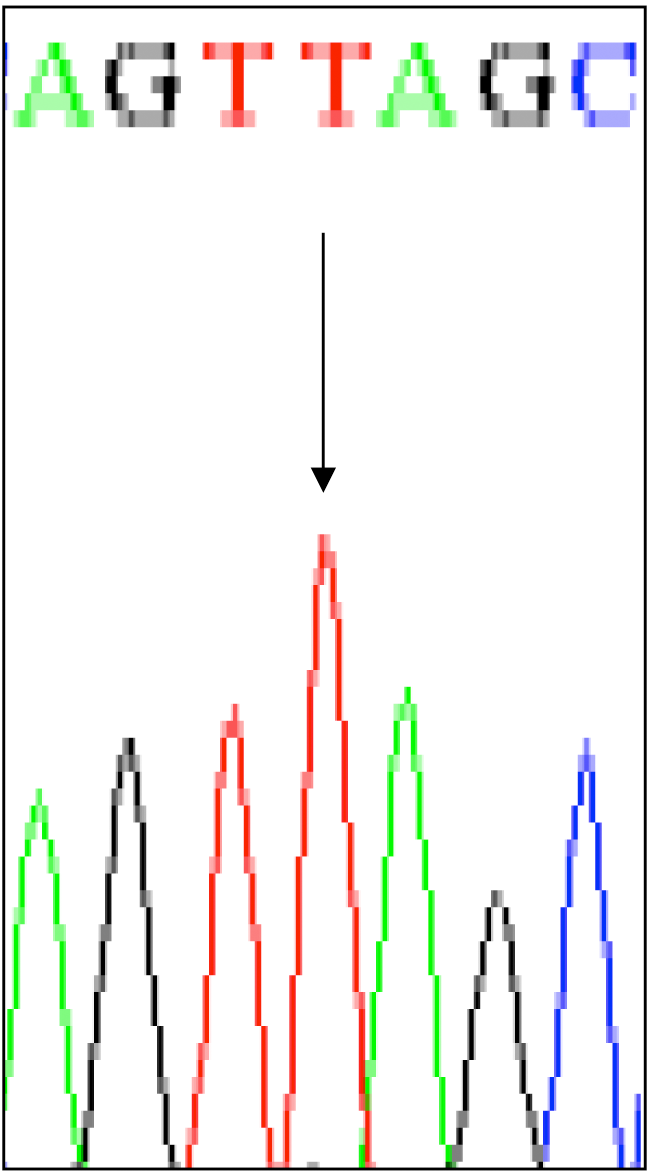

Supplement: S15 Fig — (TIF) [file pone.0115353.s015.tif]

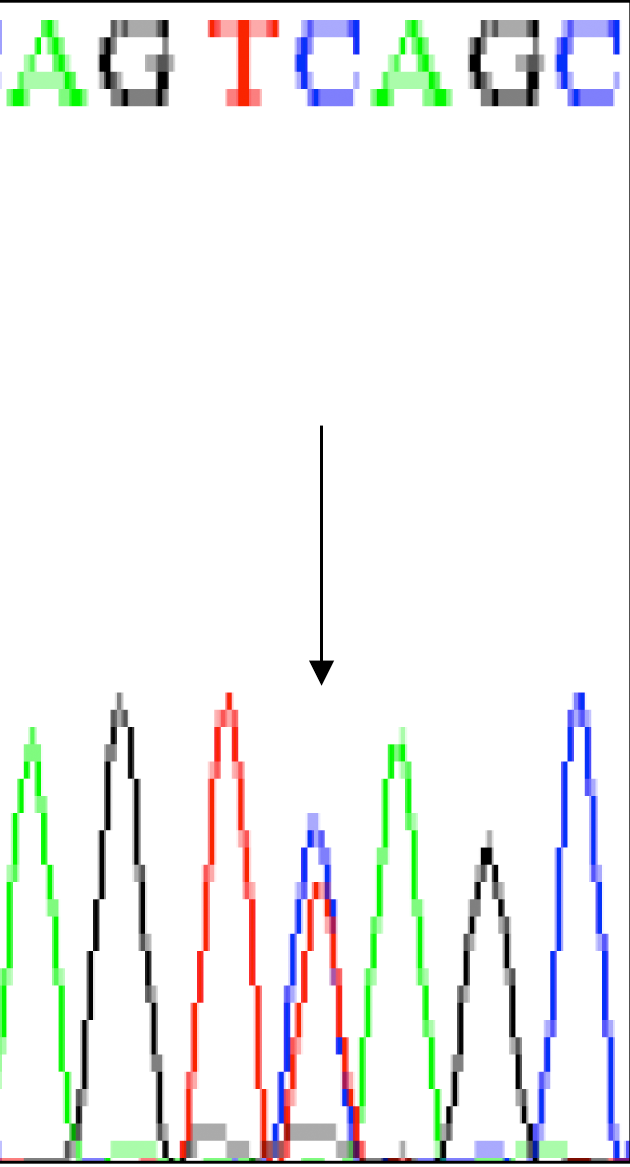

Supplement: S16 Fig — (TIF) [file pone.0115353.s016.tif]
